# The DosR regulon of *Mycobacterium avium* and adaptation to hypoxia

**DOI:** 10.3389/fcimb.2025.1545856

**Published:** 2025-02-18

**Authors:** Juan M. Belardinelli, Charlotte Avanzi, Kelsey E. Martin, Ha Lam, Marte S. Dragset, William H. Wheat, Brendan K. Podell, Mercedes Gonzalez-Juarrero, Mary Jackson

**Affiliations:** ^1^ Mycobacteria Research Laboratories, Department of Microbiology, Immunology and Pathology, Colorado State University, Fort Collins, CO, United States; ^2^ Cell and Molecular Biology, Colorado State University, Fort Collins, CO, United States; ^3^ Centre of Molecular Inflammation Research, Norwegian University of Science and Technology, Trondheim, Norway; ^4^ Department of Clinical and Molecular Medicine, Norwegian University of Science and Technology, Trondheim, Norway

**Keywords:** *Mycobacterium avium*, nontuberculous mycobacteria, DosRS, biofilm, hypoxia, virulence

## Abstract

Like other tuberculous and nontuberculous mycobacterial pathogens of human lung such as *Mycobacterium tuberculosis* and *M. abscessus*, *M. avium* is likely exposed to a variety of stressors during infection, including hypoxic conditions inside activated macrophages and in the avascular necrotic regions of granulomas. How *M. avium* survives hypoxic stress to establish a chronic infection is currently not well understood. Using RNA-sequencing, we here show that *M. avium* grown under progressive microaerophilic conditions activates more than 4-fold a subset of 16 genes, the expression of 13 of which is dependent on the two-component system regulator DosRS. A subset of *M. avium* DosR regulon genes was confirmed to also be activated upon exposure to nitric oxide. Although a second sensor kinase besides DosS has been proposed to function with the transcriptional regulator DosR in *M. avium*, we show that this other kinase cannot compensate for a deficiency in DosS. Loss of *dosRS* expression in *M. avium* led to a significant reduction in viability under hypoxia that was more marked at acidic than at neutral pH. Unlike the situation in *M. abscessus*, however, loss of DosRS did not significantly impact the ability of *M. avium* to establish a drug tolerant state *in vitro* or form biofilms under host relevant conditions. Collectively, these results are suggestive of a lesser impact of DosRS on the ability of *M. avium* to develop antibiotic tolerance compared to other nontuberculous mycobacteria. The *M. avium dosRS* mutant further showed no signs of virulence attenuation in murine macrophages and in chronically infected immunocompetent BALB/c mice.

## 1 Introduction

Nontuberculous mycobacteria (NTM) are environmental bacteria that can cause progressive, chronic and sometimes fatal pulmonary disease. Population-based investigations indicate that the prevalence of NTM-related pulmonary disease (NTM PD) continues to increase globally, with *Mycobacterium avium* complex (MAC) species and *Mycobacterium abscessus* and its subspecies (MABS) being the two most common NTM recovered from respiratory specimens (Prevots and Marras, 2015; Park and Olivier, 2015). In immunocompetent patients with predisposing lung conditions, such as cystic fibrosis (CF) and chronic obstructive pulmonary disease, the presentations of NTM PD share striking similarities with tuberculosis (TB) in immunocompetent subjects, including necrotizing and non-necrotizing granulomas and cavitation. These lesions tend to be paucibacillary with organisms seen primarily in the areas of necrosis (O’Connell et al., 2012; Tomashefski et al., 1996; Jain et al., 2017; Choi et al., 2021). These shared pathologic manifestations suggest that *Mycobacterium tuberculosis* (*Mtb*) and MAC/MABS are probably exposed to similar stresses during host infection. Whether or not *Mtb* and NTM have evolved the same strategies to overcome these stressors and establish a chronic lung infection is a question of considerable interest when designing dual anti-tuberculosis (TB)/NTM therapies.

NTM and *Mtb* are thought to share the ability to persist within lung lesions in a slow- or non-replicating state, which contributes to their drug tolerance and to treatment failure in chronically infected individuals (Wu et al., 2018). Low oxygen tension and respiratory competitors of oxygen, such as nitric oxide (NO) and carbon monoxide (CO), in avascular necrotic regions of granulomas and in the CF airway likely signal mycobacterial pathogens to adopt a non-replicating state in the absence of aerobic respiration. In *Mtb*, the initial response to hypoxia is controlled by the three-component regulatory system, DosRST. Upon activation, the transcriptional regulator DosR drives the physiologic adaptation of *Mtb* required for survival in the absence of oxygen through the induction of a 48-gene “dormancy regulon” (Park et al., 2003; Rustad et al., 2008; Leistikow et al., 2010; Reichlen et al., 2017). The finding that macaques infected with *Mtb* mutants harboring *dosR*, *dosS* and/or *dosT* null mutations displayed reduced infection levels and granulomatous inflammation during the persistent stage of infection compared to animals infected with wild-type *Mtb* supports the notion that the DosR regulon is important for persistence in a human-like model of TB infection (Mehra et al., 2015). Accordingly, inhibitors of DosRST are being sought for their potential to shorten TB treatment and lower relapse rates when used in combination with standard-of-care antibiotics (Zheng et al., 2017, 2020).

Homologs of the DosR and DosS proteins have been identified in the sequenced genomes of many *Mycobacterium* spp. as well as in a number of other environmental Actinomycetes, suggestive of a broad role of this two-component regulator in the physiologic adaptation of Actinomycetes to hypoxic conditions beyond those encountered in the infected host (Bartek et al., 2009; Gerasimova et al., 2011). Recent work by our group and others confirmed that, similar to the situation in *Mtb*, DosRS plays an important role in the adaptation of MABS to hypoxia (Belardinelli et al., 2022; Simcox et al., 2023). Importantly, our work further revealed that a deficiency in DosRS expression significantly impairs the ability of MABS to form biofilms, a phenotype likely attributable to the reduced ability of oxygen-deprived bacilli located within the biofilms to remain viable in the absence of a functional DosRS system (Belardinelli et al., 2022). Biofilm formation is a common strategy used by pathogens of the airway to colonize the host while enhancing the bacterium’s drug tolerance and resistance to host defense mechanisms, and could help explain why DosS inhibitors can reverse the tolerance of MABS bacilli to some antibiotics in a mouse model of MABS infection not known to develop hypoxic lesions (Belardinelli et al., 2022).

Like *Mtb* and MABS, MAC species are endowed with a DosRS system and further possess a second kinase (annotated as MAV_2508 in the genome of *M. avium* 104) thought to function with DosR (Gerasimova et al., 2011). In the context of our search for novel therapeutic strategies to address the growing issue of multidrug-resistant NTM infections, we here sought to experimentally characterize the DosR regulon of MAC and its activation by DosS and/or MAV_2508, and to assess its contribution to survival under hypoxia, drug tolerance, biofilm formation and pathogenicity.

## 2 Materials and methods

### 2.1 Strains and culture media

*M. avium* subsp. *hominissuis* MAH11 (Dragset et al., 2019) was grown under agitation at 37°C in Middlebrook 7H9 medium supplemented with 10% albumin-dextrose-catalase (ADC) (BD Sciences) and 0.05% Tween 80, in Synthetic CF medium (SCFM) (Belardinelli et al., 2021), or on Middlebrook 7H11 agar supplemented with 10% oleic acid-albumin-dextrose-catalase (OADC) (BD Sciences). Kanamycin and hygromycin were added to the culture media at a final concentration of 25 μg mL^-1^ and 50 μg mL^-1^, respectively. For growth under microaerophilic and hypoxic conditions, MAH11 strains were grown in Dubos-Tween albumin broth at a final pH of 7.3 or 5.7, either in standing T25 vented tissue culture flasks (microaerophilic conditions) (Zheng et al., 2017) or in 16x100 mm glass tubes with tightly sealed screw caps with rubber septa under constant stirring using Teflon-coated magnetic bars (hypoxic Wayne model) (Wayne and Hayes, 1996). Decolorization of methylene blue (1.5 μg mL^-1^ final concentration) in control tubes served as a visual indication of oxygen depletion in the Wayne model.

### 2.2 *M. avium dosRS* knock-out mutant and complemented mutant strains

A *dosRS* deletion mutant was generated in MAH11 by allelic replacement using the Ts-sacB system (Pelicic et al., 1997). Briefly, an allelic exchange substrate consisting of the kanamycin-resistance cassette (*kan*) bracketed by ~ 500 bp of upstream and downstream DNA immediately flanking the *dosRS* operon of MAH11 (genes *MAV_4109-MAV_4108* based on the *M. avium* 104 genome annotation) was cloned in pPR27xylE. The resulting plasmid, pPR27xylE-*dosRS::kan*, was electrotransformed in MAH11 and a transformant selected on kanamycin-containing agar plates at 32°C. Upon a culturing step at 32°C in liquid broth, the transformant was plated on agar with kanamycin and 2% sucrose at 39°C to select for allelic exchange mutants. Allelic replacement at the *dosRS* locus was verified by PCR and sequencing.

For complementation, 220-bp of the promoter region of *dosR* and the entire coding sequence of *dosRS* were PCR-amplified from MAH11 genomic DNA and cloned into the integrative plasmid pMV306H, yielding pMV306H-*dosRS.* Alternatively, 220-bp of the promoter region of *dosR* and the entire coding sequence of *dosR* only were PCR-amplified and cloned into the integrative plasmid pMV306H, yielding pMV306H-*dosR.* The sequences of the primers used to generate the different constructs are available upon request. A mutant carrying a transposon insertion in the second putative sensor kinase associated with DosR (gene *MAV_2508* based on the *M. avium* 104 genome annotation; position of insertion = bp 220), and a mutant carrying a transposon insertion in *dosR* (position of insertion = bp 549) were obtained from Dr. M. Dragset (Norwegian University of Science and Technology, Trondheim, Norway) (Dragset et al., 2019).

### 2.3 Biofilm assay

Biofilm formation in SCFM was monitored by crystal violet staining as described by Belardinelli et al. (2021) except that MAH11 strains were allowed to grow in poly-D-lysine-coated microtiter plates for 14 days (instead of 5 days for MABS).

### 2.4 NO susceptibility assays

For *in vitro* NO susceptibility assays, MAH11 strains and *M. abscessus* ATCC 19977 grown in 7H9 supplemented with albumin-dextrose-NaCl (ADS) and 0.05% Tween 80 to an OD_600nm_ of 0.1 were added 50 or 500 μM DETA/NO (Cayman Chemical) or Spermine/NO (Cayman Chemical) and incubated for 24 h at 37°C under agitation. Viable CFUs were enumerated by plating serial dilutions of the cell suspensions on 7H11-OADC agar.

### 2.5 Susceptibility to metronidazole, nitrofurans and other antibiotics under hypoxia

The susceptibility of MAH11 strains to metronidazole, nitrofurans and clinically used antibiotics under hypoxic conditions was determined using the Wayne model (pH 7.3) as described above. When cultures reached hypoxia (as determined by decolorization of methylene blue), metronidazole (ThermoFisher), 2-nitrofuran (Sigma-Aldrich), nitrofurazone (Sigma-Aldrich), rifampicin (Sigma-Aldrich), amikacin (Sigma-Aldrich), ethambutol (Sigma-Aldrich), clarithromycin (Sigma-Aldrich), bedaquiline or clofazimine (Sigma-Aldrich) were added with a syringe through a rubber septum, to avoid introducing oxygen. After 7 days of incubation under hypoxia at 37°C, tubes were opened and serial dilutions of the cultures plated to enumerate CFUs.

### 2.6 Metabolic labeling of lipids

Metabolic labeling of MAH11 cells with [1,2-^14^C]acetic acid (0.5 μCi mL^-1^; specific activity, 54.3 Ci/mol, PerkinElmer) was performed for 16 h at 37°C in Dubos-Tween albumin broth under microaerophilic conditions in standing T25 vented flasks, or under well aerated conditions in Erlenmeyer flasks. [1,2-^14^C]acetic acid-derived lipids extracted from whole bacterial cells with a mixture of chloroform and methanol (1:2 and 2:1, by vol.) were analyzed by thin-layer chromatograph (TLC) on aluminum-backed silica gel 60-precoated plates F254 (E. Merck) and revealed by PhosphorImaging.

### 2.7 RNA extraction and RT-qPCR

Two independent cultures of bacteria grown under microaerophilic conditions in Dubos-Tween albumin broth (or aerophilic conditions for 24 h) were used for transcriptomics analyses. RNA extraction with the Direct-zol™ RNA Miniprep kit (Zymo Research), reverse transcription reactions using the Superscript IV First-Strand Synthesis System (Thermo Fisher) and RT-qPCR using the SYBR Green PCR Master Mix (Sigma-Aldrich) were conducted as per the manufacturers’ protocols and analyzed on a CFX96 real-time PCR machine (Bio-Rad). PCR conditions: 98°C (30 s; enzyme activation), followed by 40 cycles of 98°C (10 s; denaturation) and 60°C (30 s; annealing/extension). Mock reactions (no reverse transcription) were done on each RNA sample to rule out DNA contamination. The target cDNA was normalized internally to the *sigA* cDNA levels in the same sample. For RT-qPCR of MAH11-infected macrophages, infections were conducted as described below and, at the indicated time points, 1 mL TRIzol reagent (Thermo Fisher) was added to triplicate wells. Cells were scraped, and RNA extracted and processed as described above. The following primers were used: sigA Fw (5’-CCTACCTCAAGCAGATCGGT-3’); sigA Rv (ATCTCCGACATCAGCTGGG); dosR Fw (5’-GATGCTGACGTCGTTCACC-3’); dosR Rv (5’-TCCATGCCCTTGATGTCCTT-3’); MAV_1793 Fw (5’-GCCCAAGGACCTGACTAACC-3’); MAV_1793 Rv (5’-TCCACTCCTTGAACTTCGCC-3’); MAV_2505 Fw (5’ATGCAAATGACCGCGGATAC-3’); MAV_2505 Rv (5’-GATTTCACTGTTCGGCGCG-3’); MAV_2507 Fw (5’TTGCTCGGTTCGGTCAGTTC-3’); and MAV_2507 Rv (5’-GGTAGGGCATCATCGGATCC-3’).

### 2.8 RNAseq library preparation and data analysis

RNA-seq libraries preparation and data processing was conducted as described previously (Belardinelli et al., 2022). Gene expression and differential expression analysis was completed in R (version 3.6.0) using DESeq2 (version 1.26.0) (Love et al., 2014). Genes were identified as differentially expressed if they had a log_2_ fold change greater than 2 and a Benjamini-Hochberg multiple testing correction adjusted *P*-value of 0.05 or less. Venn diagrams were designed and analyzed using InteractiVenn (Heberle et al., 2015).

### 2.9 Data availability

The sequencing data described in this publication have been deposited in the NCBI Sequence Read Archive (SRA) under accession number PRJNA1191895, https://www.ncbi.nlm.nih.gov/bioproject/PRJNA1191895.

### 2.10 Macrophage infections

Murine RAW 264.7 cells were grown in Dulbecco’s modified Eagle medium (DMEM) (Corning) supplemented with 10% fetal bovine serum and 1% penicillin-streptomycin and seeded in 24-well or 96-well plates for RNA extraction and CFU enumeration, respectively. After a 24 h incubation period to allow the cells to attach to the wells, cells were washed with warm PBS and triplicate wells were infected with well-dispersed suspensions of the WT and mutant strain in antibiotic-free DMEM at an MOI of 1 for 2 h at 37°C. Cells were then washed three times with warm PBS and the wells were replenished with DMEM containing 250 μg mL^-1^ amikacin to kill extracellular bacteria. After an hour of incubation, cells were washed three more times with PBS and incubated in DMEM for the remainder of the experiment. Two, 24, 48, 72 and 120 hours post-infection, viable intracellular bacteria were assessed by lysing the cells in sterile water containing 0.1% Triton X-100, and plating serial dilutions on 7H11-ADC agar plates to enumerate CFUs.

### 2.11 Mouse infections

All protocols and use of these animals were approved by the Institutional Animal Care and Use Committee (IACUC) at Colorado State University. Studies were performed in accordance with recommendations of the Guide for the Care and Use of Laboratory Animals of the National Institutes of Health.

Six to eight-week-old female BALB/c mice were purchased from Jackson laboratories. Animals were rested for a week, weighed, and divided into randomized groups for the study. Mice were infected with fresh cultures of MAH11 WT and MAH11Δ*dosRS* (grown to OD_600 nm_ 0.6 – 0.8 in 7H9-ADC-Tween 80). To this end, mice were anesthetized with a mix of isoflurane and oxygen (1.5- 2% at a flow rate of 0.4-0.8 L/min) and two doses of fifty microliters each of inoculum were delivered intratracheally as an intrapulmonary spray instillation to each animal using a high-pressure syringe device (PennCentury), for a targeted dose of 1x10^6^ CFU/lung. To confirm the actual bacterial deposition in the lungs, mice (n=5) were sacrificed 16 h after instillation and the whole lung prepared for viable bacteria quantification. Tissues were homogenized using the Precellys Tissue Homogenizer (Precellys Lysing Kit, 220325-830) and serial dilutions of each homogenate were applied to 7H11-OADC agar supplemented with carbenicillin (Sigma-Aldrich) and cycloheximide (GoldBio). The plates were cultured for 2-3 weeks at 37°C until visible CFUs could be enumerated. Following infection, the mice were monitored daily for indications of weight loss or abnormal behavior requiring pre-endpoint euthanasia. The remaining groups of mice (n=5) were euthanized at each defined timepoint, and the left lobe lung and spleen were enumerated for CFUs while the right lung lobe was fixed and permeabilized in 4% *para*-formaldehyde (PFA) for staining and histological analysis. Mice were euthanized via narcosis with CO_2_ (5.8L, flow rate of 3.0 (1.7-4) L/min). Standard histological protocols for sectioning and staining with Hematoxylin-Eosin (H&E) and Ziehl-Neelsen acid fast stain were used. Slide were scanned at 40X magnification using a multispectral automated PhenoImager (Akoya Biosciences) and analyzed as described previously (Cooper et al., 2024), and final edits performed by the reviewing pathologist.

### 2.12 Statistical analysis

Statistical tests were performed as indicated in the figure legends. Calculations were performed using Graphpad Prism version 9.5.1 for Windows (San Diego California USA).

## 3 Results

### 3.1 Transcriptional response of MAC to growth under microaerophilic conditions

The *M. avium* complex consists of a growing number of species as described in recent reviews (Daley, 2017; Busatto et al., 2019). However, the three most important human pathogens are *M. avium*, *M. intracellulare* and *M. chimaera.* Of these, *M. avium* subsp. *hominissuis* is the most frequent species isolated from patients with pulmonary infections and was thus chosen for the purpose of this study. Isolate MAH11 was chosen for its amenability to genetic manipulations, genome sequence availability, and ability to establish an infection in the lungs of mice (Dragset et al., 2019).

**Figure 1:**
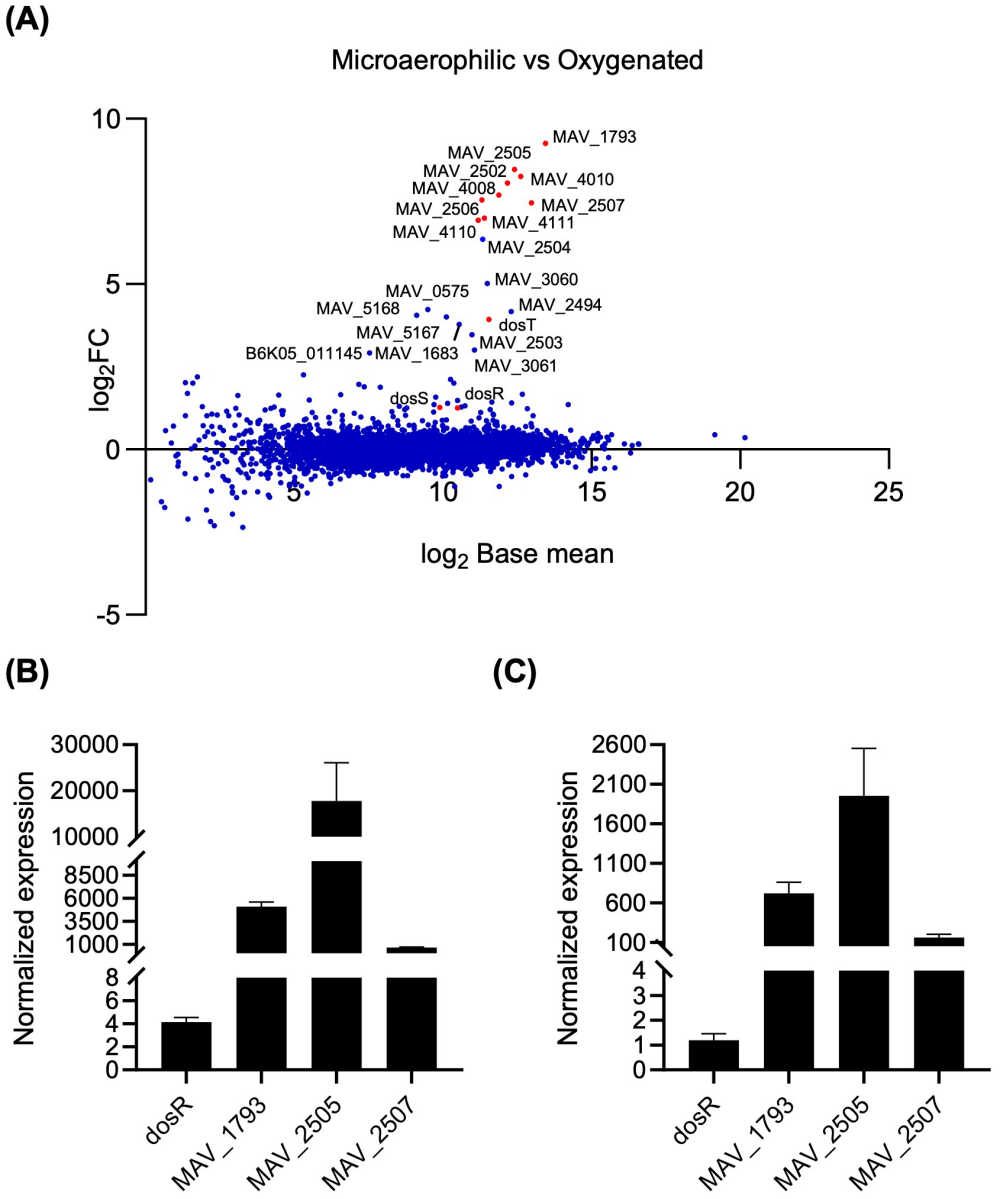
Transcriptional response of wild-type *M. avium* MAH11 to oxygen depletion. **(A)** Differentially expressed genes in WT *M. avium* MAH11 grown in Dubos-Tween albumin broth for 24 h under microaerophilic conditions in standing T25 vented tissue culture flasks compared to well-aerated shaking flasks (oxygenated). Values are expressed as log_2_-fold change (FC) versus log_2_ base mean. Positive log_2_FC values indicate upregulation under microaerophilic conditions. Genes predicted bioinformatically to belong to the DosRS regulon of *M. avium*, including *dosR* and *dosS*, are shown in red (Gerasimova et al., 2011). **(B)** RT-qPCR showing the upregulation of *dosR* and the *MAV_1793*, *MAV_2505* and *MAV_2507* genes in WT MAH11 grown under microaerophilic conditions compared to oxygenated conditions as described above. cDNA was normalized internally to *sigA* cDNA in the same samples. For each gene, cDNA levels under microaerophilic conditions are expressed relative to cDNA levels under oxygenated conditions arbitrarily set to 1. The results shown are means ± SD of biological triplicates (n = 3 RNA extractions and RT-qPCR reactions). **(C)** RT-qPCR showing the upregulation of *MAV_1793*, *MAV_2505* and *MAV_2507* in WT MAH11 grown in 7H9-ADS-Tween 80 to an OD_600nm_ of 0.2 and treated with 500 μM DETA/NO for 1 h. cDNA was normalized internally to *sigA* cDNA in the same samples. cDNA levels in NO-treated samples are expressed relative to cDNA levels measured in the untreated control arbitrarily set to 1. The results shown are means ± SD of biological triplicates (n = 3 RNA extractions and RT-qPCR reactions).

RNAseq transcriptional profiling was used to determine differential gene expression between wild-type (WT) *M. avium* subsp. *hominissuis* MAH11 grown for 16 h in Dubos-Tween-albumin broth under well-aerated (normoxic) conditions and microaerophilic conditions (**Figure 1A**). The list of differentially expressed (DE) genes (log_2_ fold change (FC) > 2 with a false discovery rate adjusted *P* < 0.05), which is presented in **Table 1**, indicated that 16 genes were upregulated under microaerophilic conditions. Eleven of them were upregulated more than 5 log_2_-fold. No genes were found to be significantly downregulated per the log_2_ FC and Padj cut-off values set for this study. Upregulated genes mapped to seven different locations of the *M. avium* genome with the largest cluster of induced genes encompassing *MAV_2494* through *MAV_2508* (based on the *M. avium* 104 genome annotation). The products of these genes included four universal stress family proteins (USPs), three NAD(P)H nitroreductases, a sensor histidine kinase (MAV_2508) previously proposed to function with DosR (Gerasimova et al., 2011), a putative fatty acyl desaturase, a truncated hemoglobin, oxidoreductases, and hypothetical proteins of unknown function (**Table 1**). *dosS* (*MAV_4108*) and *dosR* (*MAV_4109*) were not part of the list despite the finding of a putative DosR-binding motif in the promoter region of *M. avium dosR* suggestive of autoregulation. RT-qPCR analyses conducted on three arbitrarily chosen DE genes (*MAV_1793*, *MAV_2505* and *MAV_2507*) confirmed the RNAseq results, showing a 630 to 17,800-fold upregulation of these genes, and revealed a comparatively mild (~ 4-fold) induction of *dosR* under microaerophilic conditions (**Figure 1B**). The same three upregulated genes under microaerophilic conditions were also induced ~ 160 to 1,954-fold upon exposure of *M. avium* MAH11 to NO (**Figure 1C**). *dosR* expression, in contrast, was not significantly induced by NO (**Figure 1C**).

**Table 1:** DosRS-dependent and -independent response of *M. avium* MAH11 to microaerophilic conditions.

MAH11 gene	MAC104	Annotation	Predicted DosR binding site?^c^	log_2_ FC in microaerophilic vs oxygenated WT MAH11	log_2_ FC microaerophilic (vs MAH11 WT)			*Mabs* ATCC19977 homolog	In *Mabs* DosR regulon?^a^	*Mtb* H37Rv homolog	In *Mtb* DosR regulon?^b^
					Δ*dosRS*	Δ*dosRS* + *dosR*	Δ*dosRS* + *dosRS*				
Genes induced under microaerophilic conditions											
B6K05_002630	MAV_0575	Conserved hypothetical protein	No	4.23	0.38	1.54	-0.08	**─**	No	**─**	No
B6K05_007890	MAV_1683	Hypothetical protein	No	3.78	1.20	2.36	0.08	**─**	No	**─**	No
**B6K05_008425**	**MAV_1793**	**Fatty acid desaturase**	Yes	9.25	-8.61	-7.15	0.60	MAB_3354	Yes	─	No
**B6K05_010965**	**MAV_2494**	**Universal stress protein family protein**	No	4.16	-4.23	-4.13	0.79	─	No	Rv2005c	Yes
**B6K05_011005**	**MAV_2502**	**NAD(P)H nitroreductase**	Yes	8.06	-8.56	-8.22	0.94	─	No	Rv3131	Yes
**B6K05_011010**	**MAV_2503**	**Hypothetical protein**	No	3.47	-3.08	-3.21	0.76	MAB_3442	No	─	No
**B6K05_011015**	**MAV_2504**	**Hypothetical protein**	No	6.36	-6.73	-5.92	0.66	─	No	─	No
**B6K05_011020**	**MAV_2505**	**NAD(P)H nitroreductase acg**	Yes	8.46	-8.48	-7.15	0.58	MAB_3903	Yes	Rv2032	Yes
**B6K05_011025**	**MAV_2506**	**Universal stress protein family protein**	Yes	7.54	-8.27	-6.72	0.61	─	No	Rv2026c	No
**B6K05_011030**	**MAV_2507**	**Universal stress protein family protein**	Yes	7.46	-7.67	-6.98	0.79	MAB_2489	Yes	Rv2005c	Yes
**B6K05_011035**	**MAV_2508**	**Putative sensor kinase**	Yes	3.93	-3.94	-3.83	0.62	─	No	Rv2027c (dosT)	No
B6K05_012900	MAV_3060	DUF4383 domain-containing protein	No	5.02	0.05	0.82	-0.13	**─**	No	**─**	No
**B6K05_017915**	**MAV_4008**	**Group III truncated hemoglobin**	Yes	7.69	-8.14	-7.73	0.38	─	No	─	No
**B6K05_017920**	**MAV_4010**	**Universal stress protein family protein**	Yes	8.25	-8.60	-7.55	0.55	MAB_3904	Yes	Rv3134c	Yes
**B6K05_018420**	**MAV_4110**	**Pyridoxamine 5’-phosphate oxidase family protein**	Yes	6.93	-3.63	-3.42	0.31	MAB_1041	No	─	No
**B6K05_018425**	**MAV_4111**	**Nitroreductase**	Yes	6.99	-7.28	-7.25	0.27	─	No	Rv3131	Yes
Genes downregulated in MAH11Δ*dosRS* vs WT MAH11											
B6K05_008010	─	Short peptide	No	-0.05	-4.16	-3.20	-3.66	─	No	─	No
B6K05_008015	MAV_1709	Class A beta-lactamase-related serine hydrolase	No	-0.31	-4.15	-4.01	-4.23	MAB_0853	No	Rv2463 (lipP)	No
B6K05_008020	MAV_1710	tRNA-Gly	No	-0.13	-2.74	-3.66	-3.31	MAB_t5025c	No	glyV	No
B6K05_009065	MAV_2101	Aldehyde dehydrogenase	No	0.13	-3.66	-3.71	-3.59	MAB_4484	No	Rv0223c	No
B6K05_009340	MAV_5024	DEAD/DEAH box helicase	No	0.26	-5.06	-5.05	-4.99	─	No	Rv2024c	No

**Figure 2:**
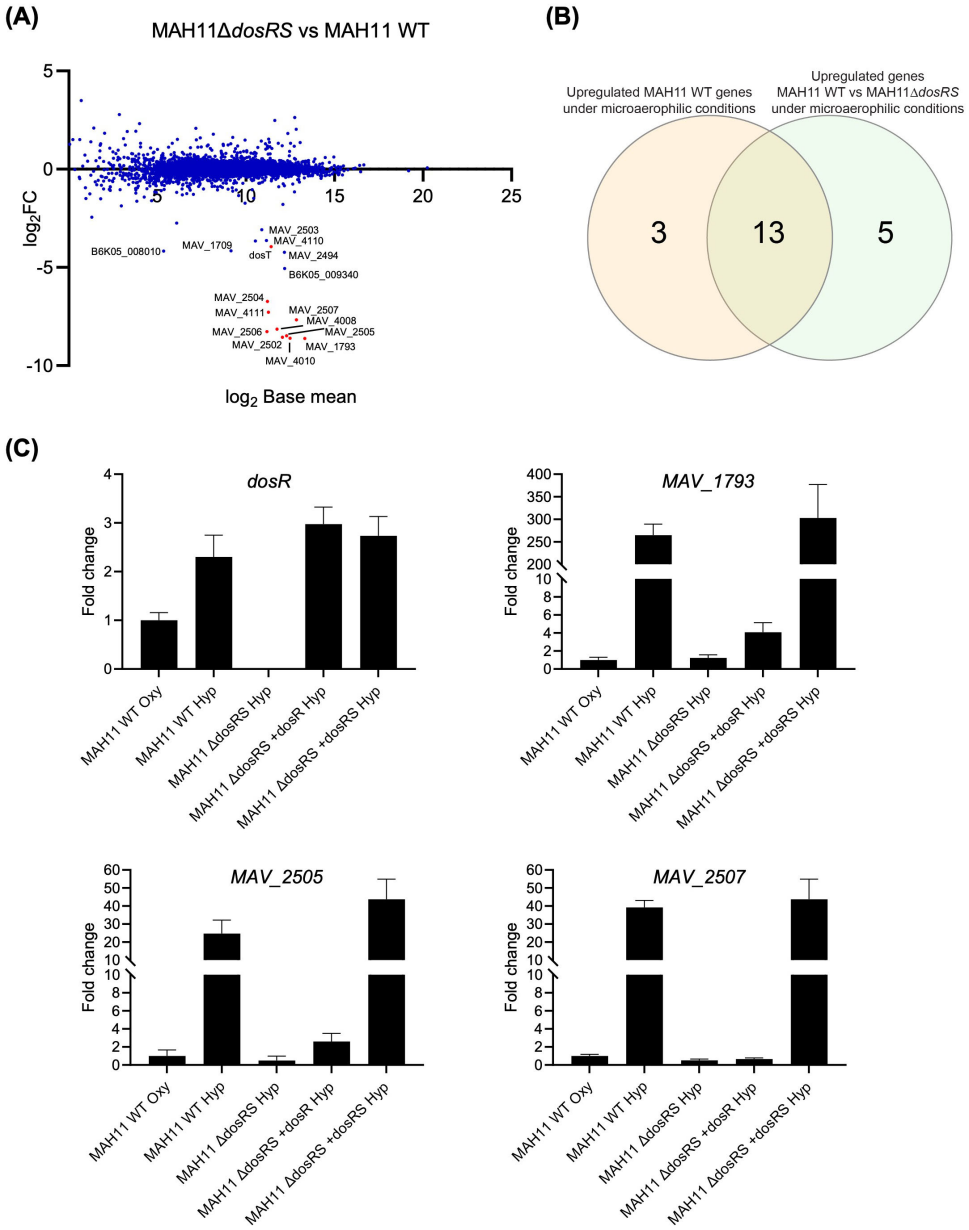
Effect of disrupting *dosRS* on the transcriptional profile of *M. avium* MAH11 under microaerophilic conditions. **(A)** Differentially expressed genes comparing *M. avium* MAH11 WT to MAH11Δ*dosRS* grown in Dubos-Tween albumin broth for 24 h under microaerophilic conditions in standing T25 vented tissue culture flasks. Values are expressed as log_2_FC versus log_2_ base mean. Negative log_2_FC values indicate reduced expression in MAH11Δ*dosRS* compared to WT MAH11. Genes predicted bioinformatically to belong to the DosRS regulon of *M. avium* (not including *dosR* and *dosS*) are shown in red (Gerasimova et al., 2011). **(B)** Venn-diagram showing the overlap between genes induced under microaerophilic conditions in WT MAH11 (from **Figure 1A**; log_2_FC > 2, padj < 0.05) and genes downregulated in MAH11Δ*dosRS* under microaerophilic conditions from **(A)** (log_2_FC < -2, padj < 0.05). **(C)** An aliquot of the RNA used for RNA-seq in **(A)** was reverse transcribed to cDNA followed by qPCR of *dosR* and DosRS regulon genes, *MAV_1793*, *MAV_2505* and *MAV_2507*. All cDNAs were normalized internally to *sigA* cDNA. Results are expressed as fold changes over the level of expression of the same genes under well-aerated conditions (“oxy”). The results shown are means ± SD of biological triplicates (n = 3 RNA extractions and RT-qPCR reactions).

The 16 DE genes in WT *M. avium* MAH11 grown under microaerophilic vs normoxic conditions (from **Figure 1A**), and the 18 DE genes in MAH11Δ*dosRS* vs WT MAH11 grown under microaerophilic conditions (from **Figure 2A**) are listed, along with the name of their *M. abscessus* (*Mabs*) and *M. tuberculosis* (*Mtb*) homolog, if applicable. The 13 common DE genes to both datasets are in bold. ^a^ – Belardinelli et al. (2022); ^b^ – Park et al. (2003); ^c^ – Gerasimova et al. (2011).

### 3.2 Role of DosRS in the transcriptional adaptation of *M. avium* to anaerobiosis

With the goal to determine how the loss of a functional DosRS regulatory system might impact the transcriptional response of *M. avium* to oxygen depletion, a *dosRS* knock-out mutant was generated by allelic replacement in MAH11 (**Supplementary Figure S1**), and the gene expression of the mutant under microaerophilic conditions was compared to that of the similarly grown WT parent using RNAseq transcriptional profiling. Two complemented mutants, one fully complemented with rescue copies of *dosR* and *dosS*, and one solely rescued with *dosR*, were included in the study.

In the absence of DosRS, 18 genes were expressed at lower levels relative to WT MAH11 (log_2_ fold change > 2 with a false discovery rate adjusted *P* < 0.05) (**Figure 2A**; **Table 1**). Thirteen of these genes were identical to those reported to be upregulated in WT MAH11 grown under microaerophilic conditions compared to normoxic conditions (**Figure 2B**; **Table 1**). We conclude from these results that the upregulation of these 13 genes in response to oxygen depletion is directly or indirectly dependent on the two-component regulator DosRS. No genes were expressed at higher levels in the mutant relative to the WT strain (per the log_2_ FC and Padj cut-off values set for this study) suggesting that *M. avium* DosRS solely acts as an activator under microaerophilic conditions. Complementation of *dosRS* knock-out with a WT copy of the *dosRS* genes restored WT gene expression in the mutant (**Table 1**). In contrast, complementation of the mutant with the sole *dosR* gene did not restore the expression of any of the 13 genes (**Table 1**). It follows that the sensor kinase encoded by *dosS* is required for the upregulation of these genes under microaerophilic conditions, and that no other sensor kinase can compensate for its activity. While the putative sensor histidine kinase encoded by *MAV_2508* has previously been proposed to function with *M. avium* DosR (Gerasimova et al., 2011), an analysis of its primary sequence indicated that it is in fact devoid of a functional heme-binding (GAF-A) domain (Sivaramakrishnan and de Montellano, 2013) (data not shown). Collectively, these results thus exclude a participation of MAV_2508 in the DosR-mediated response of *M. avium* to hypoxia. RT-qPCR analyses conducted on three DE genes (*MAV_1793*, *MAV_2505* and *MAV_2507*) confirmed the RNAseq results (**Figure 2C**).

A prior bioinformatics study identified a putative DosR-binding motif in the promoter region of 12 *M. avium* 104 genes (Gerasimova et al., 2011). Ten of the 13 DosRS-dependent DE genes identified in our RNAseq study harbored this DosR-binding motif in their promoter (**Table 1**). In contrast, none of the 5 genes found to be differentially expressed between WT MAH11 and MAH11Δ*dosRS*, but not between normoxic vs microaerophilic WT MAH11 (**Figure 2B**), harbored this motif (**Table 1**). Moreover, WT expression of these five genes was not restored in MAH11Δ*dosRS* upon complementation with *dosRS* raising doubts as to their direct control by the two-component system regulator (**Table 1**).

In line with the fact that no triglyceride synthase gene appears to be under control of DosRS in MAH11 (**Figure 2A**; **Table 1**) [in contrast to the situation in *Mtb* (Rustad et al., 2008)], the metabolic labeling of WT MAH11, MAH11Δ*dosRS* and the two complemented mutants with [1,2-^14^C]acetate failed to reveal any differences in the *de novo* synthesis of triglycerides under microaerophilic conditions between the four strains (**Supplementary Figure S2**).

### 3.3 Effect of DosRS inactivation on the viability of MAH11 under hypoxic conditions

**Figure 3:**
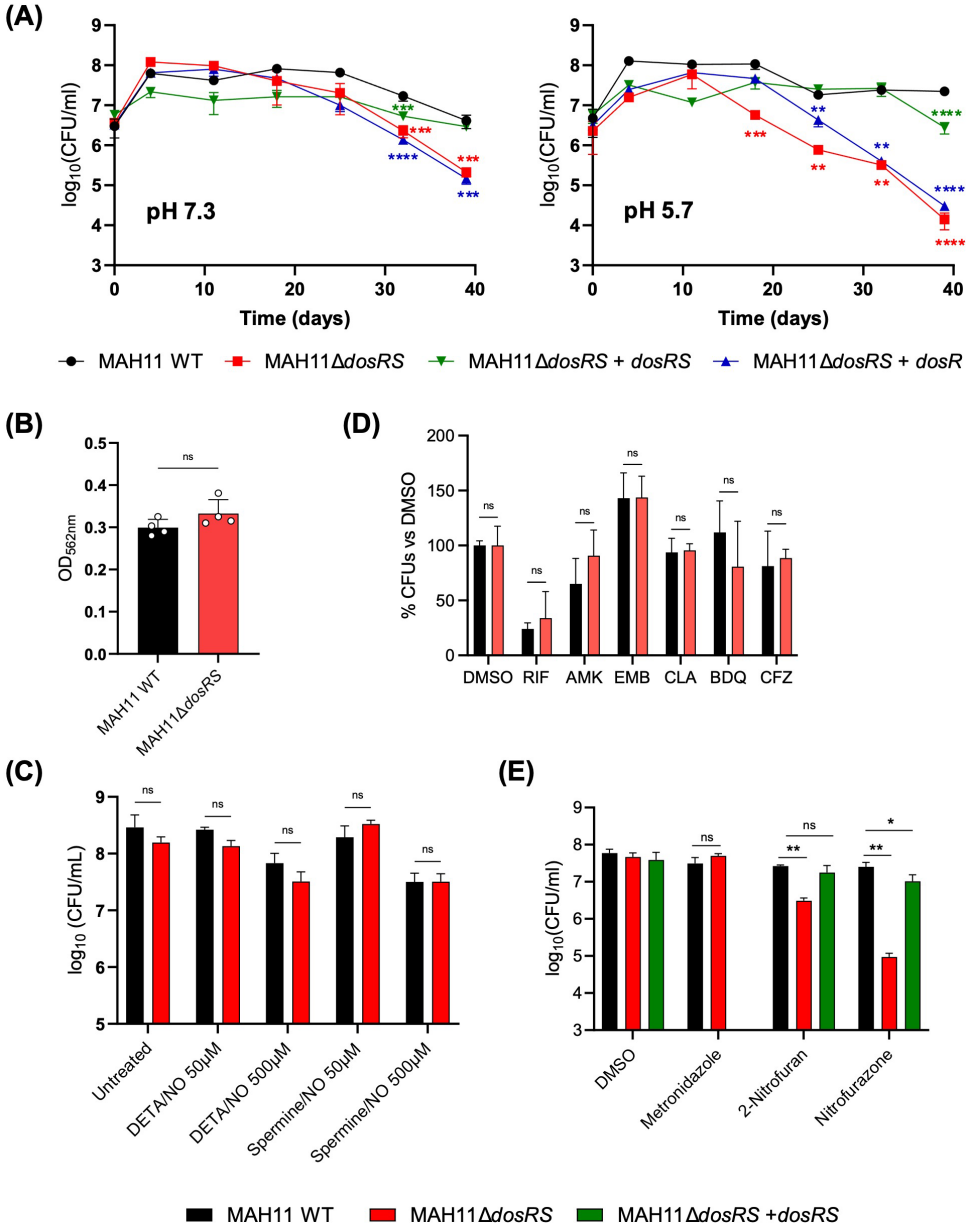
Phenotypic characterization of the *M. avium dosRS* knock-out mutant. **(A)** Comparative survival curves of MAH11 WT, MAH11Δ*dosRS* and MAH11Δ*dosRS* complemented with *dosR* or *dosRS* in the Wayne model at pH 7.3 (left) and 5.7 (right). At the indicated time points, tubes were opened and serial dilutions of the cultures plated for CFU enumeration. The results presented are the means ± SD of technical triplicates. Asterisks denote statistically significant differences relative to WT MAH11 pursuant to Dunnett’s one-way ANOVA, with **p<0.005; ***p<0.0005; and ****p<0.0001. **(B)** Biofilm formation of MAH11 WT and MAH11Δ*dosRS* after 14 days of incubation in SCFM in poly-D-lysine-coated microplates was determined by crystal violet staining. Crystal violet absorbed by the biofilm matrix was extracted with 300 μL of 30% acetic acid for 30 min followed by reading of the absorbance of the solution at 562 nm. The values reported on the Y-axis are the means ± SD of four biological replicates. “ns” indicates that the difference between the two strains is not significant pursuant to Dunnett’s one-way ANOVA. **(C)** Susceptibility of *M. avium* MAH11 WT and MAH11Δ*dosRS* to NO. Triplicate cultures of WT MAH11 and MAH11Δ*dosRS* grown in 7H9-ADS-Tween 80 were either untreated or treated with 50 or 500 μM DETA/NO or Spermine/NO for 24 h and subsequently plated for CFU enumeration. The values reported on the Y-axis are the means ± SD of three technical replicates. “ns” indicates that the difference between the two strains is not significant pursuant to Dunnett’s one-way ANOVA. The susceptibility of MAH11 strains to clinically used antibiotics (RIF, rifampicin; AMK, amikacin; EMB, ethambutol; CLA, clarithromycin; BDQ, bedaquiline; CFZ, clofazimine) **(D)** and metronidazole and nitrofurans **(E)** under hypoxic conditions was determined using the Wayne model. Upon onset of anaerobiosis (as determined by decolorization of methylene blue), drugs were added and the cultures incubated for another 7 days. At the end of the incubation time, serial dilutions of the cultures were plated to enumerate CFUs. The values reported on the Y-axis are the means ± SD of technical triplicates. Asterisks denote statistically significant differences relative to WT MAH11 pursuant to Dunnett’s one-way ANOVA, with **p<0.05; ***p<0.0005; and ****p<0.0001; ns, not significant. The results shown in **(A)** through **(E)** are representative of three independent experiments.

To next determine whether the inability of the *dosRS* mutant to induce a subset of genes in response to oxygen depletion negatively impacted the survival of MAH11 under hypoxia, we resorted to the well-established Wayne model to culture MAH11 for up to 35 days under hypoxic conditions, and monitored the viability of the WT, mutant and complemented mutant strains over time. This experiment was conducted at both pH 7.3 and pH 5.7 because acidic conditions, which prevail in the phagolysosome of activated macrophages, have been shown to drastically decrease the anaerobic survival of an *Mtb dosR* mutant (Reichlen et al., 2017). The results, which are shown in **Figure 3A**, revealed a significant survival defect of the MAH11 *dosRS* mutant at both pHs, though the defect was more pronounced at acidic pH. WT survival was restored in the mutant complemented with *dosRS* but not with *dosR* only, highlighting the critical role of DosS in the ability of *M. avium* to sense and respond to hypoxic stress.

Consistent with the apparent lack of involvement of MAV_2508 in the hypoxic response of *M. avium*, an MAH11 mutant harboring a transposon insertion in this gene did not show any survival defects in the same hypoxic model (**Supplementary Figure S3**).

### 3.4 Effect of DosRS inactivation on biofilm formation

The impact of DosRS on the ability of *M. avium* to form biofilms was tested in our recently developed synthetic CF medium (SCFM) model (Belardinelli et al., 2021). A comparative assessment of the ability of the WT, *dosRS* mutant and complemented mutant strains to form biofilms in SCFM-containing poly-D-lysine-coated microtiter plates revealed no significant differences between strains (**Figure 3B**).

### 3.5 Impact of DosRS on the resistance of *M. avium* to NO

To next determine whether DosRS enhances the resistance of *M. avium* to NO, WT MAH11 and MAH11Δ*dosRS* cells were exposed to 50 or 500 μM of DETA/NO and spermine/NO for 24 h and viability was subsequently assessed by CFU plating. CFU counts revealed no difference in susceptibility to NO between the two strains (**Figure 3C**). Of note, *M. avium* MAH11 demonstrated a high-level intrinsic resistance to NO. Indeed, exposure to 500 μM of DETA/NO or spermine/NO for 24 h only resulted to in a 0.6 to 1 log_10_ decrease in viable MAH11 CFUs, whereas a reduction of 2 to 2.8 log_10_ CFUs was observed with *M. abscessus* (**Supplementary Figure S4**).

### 3.6 Impact of DosRS on drug tolerance *in vitro*

In *M. abscessus*, DosRS is important to the development of drug tolerance under early anaerobic dormancy (Belardinelli et al., 2022). To determine if this attribute of the DosRS two-component regulator also applied to *M. avium*, WT MAH11 and the *dosRS* knock-out mutant grown under hypoxic conditions were treated for 7 days with either DMSO (negative control) or the clinically relevant antibiotics, rifampicin, amikacin, ethambutol, clarithromycin, bedaquiline and clofazimine. Enumeration of surviving bacteria post-treatment indicated that the WT and mutant strains were both fully tolerant to almost all antibiotics tested (**Figure 3D**). Only rifampicin showed significant killing in the Wayne model, but WT and mutant strains did not significantly differ in their tolerance to this drug (**Figure 3D**). We conclude from this experiment that *dosRS* does not play a significant role in the ability of *M. avium* to develop tolerance to clinically used antibiotics under hypoxia.

Metronidazole and nitrofurans are classes of drugs that require partial reduction at their nitro groups by dedicated NAD(P)H nitroreductases to generate highly reactive, bactericidal, intermediates. Since as many as three NAD(P)H nitroreductase genes were found to be under control of DosRS in *M. avium* (**Table 1**), we set out to determine whether the lack of induction of these genes in the *dosRS* knock-out mutant under hypoxia enhanced the level of resistance to metronidazole, nitrofurazone and 2-nitrofuran. While the mutant displayed WT susceptibility to metronidazole, we found that it was significantly more susceptible to both nitrofurans (**Figure 3E**). WT susceptibility to nitrofurans was restored in the *dosRS* complemented mutant.

### 3.7 Impact of DosRS on the survival of *M. avium* inside macrophages

**Figure 4:**
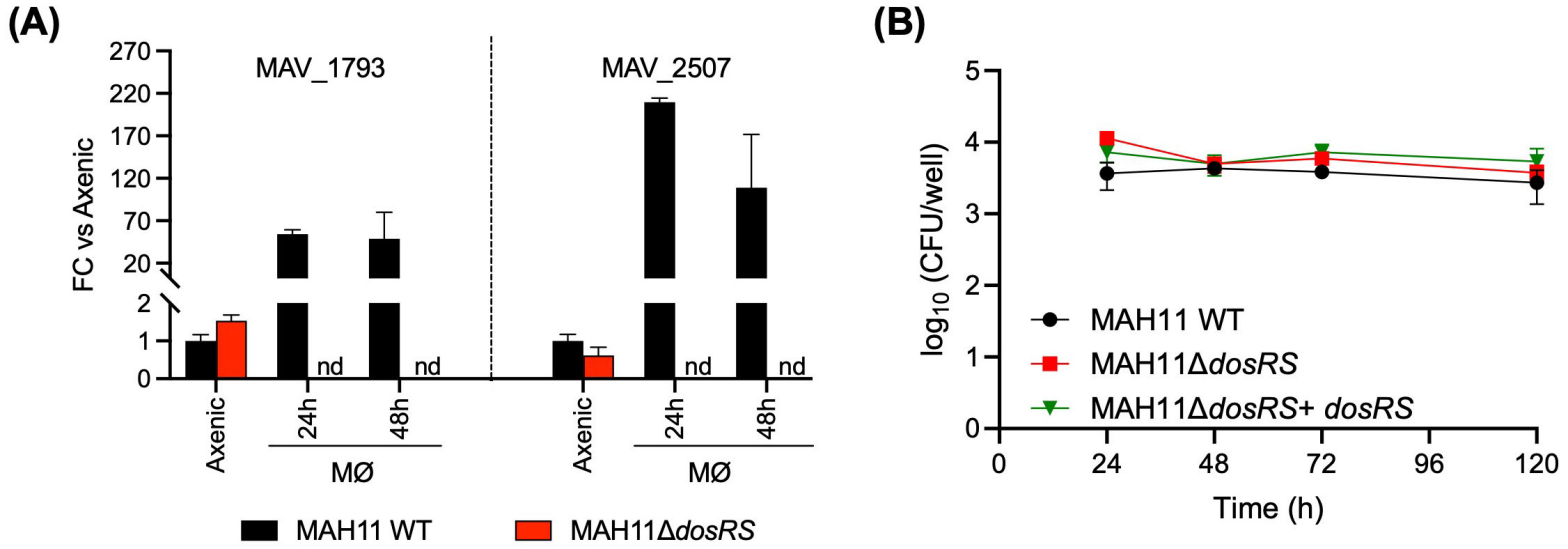
Role of DosRS on the survival of *M. avium* inside macrophages. **(A)** Evidence of DosRS regulon induction upon macrophage infection. The expression of DosRS regulon genes, *MAV_1793* and *MAV_2507*, following infection of RAW 264.7 cells with WT MAH11 or MAH11Δ*dosRS* was monitored by RT-qPCR over time. RAW cells were infected for 2 h at an MOI of 1, followed by a 1 h-treatment with amikacin to eliminate extracellular bacteria. After this treatment (defined as time 0), cells were collected 24 and 48 h post-infection, and RNA extracted and processed for RT-qPCR analyses as described under Materials and Methods. Results are expressed as fold changes over the level of expression of the same genes in WT MAH11 grown to OD_600 nm_ 0.2 in Dubos-Tween 80-albumin under well aerated conditions (labeled as “axenic” on the graph). The results were standardized to *sigA* expression levels in the same samples and are shown as means ± SD of biological triplicates (n = 3 RNA extractions and RT-qPCR reactions). nd, not detected. **(B)** RAW 264.7 cells were infected with either WT MAH11, MAH11Δ*dosRS* or MAH11Δ*dosRS* complemented with *dosRS* at an MOI of 1. At the indicated time points, cells were lysed and lysates plated on 7H11-OADC agar for CFU enumeration. Shown are the means ± SD of triplicate wells.

To investigate potential effects of DosRS on the interactions of *M. avium* with macrophages, RAW 264.7 cells were infected with WT MAH11, the *dosRS* mutant and the *dosRS* complemented mutant, and the different strains compared for their intracellular replication and survival. Macrophages were infected at an MOI of 1. Two DosR regulon genes, *MAV_1793* and *MAV_2507*, were found to be strongly induced in WT *M. avium* MAH11 (but not in the *dosRS* knock-out mutant) residing intracellularly, indicating that DosRS is induced upon macrophage infection (**Figure 4A**). Determination of intracellular CFUs 1, 2, 3 and 5 days post-infection revealed comparable infection kinetics for the three strains (**Figure 4B**). Thus, despite being induced intracellularly, the two-component regulatory system DosRS is not critical to the survival of *M. avium* inside macrophages under the conditions of this study.

### 3.8 Virulence of a *dosRS* knock-out mutant in a murine model of *M. avium* infection

**Figure 5:**
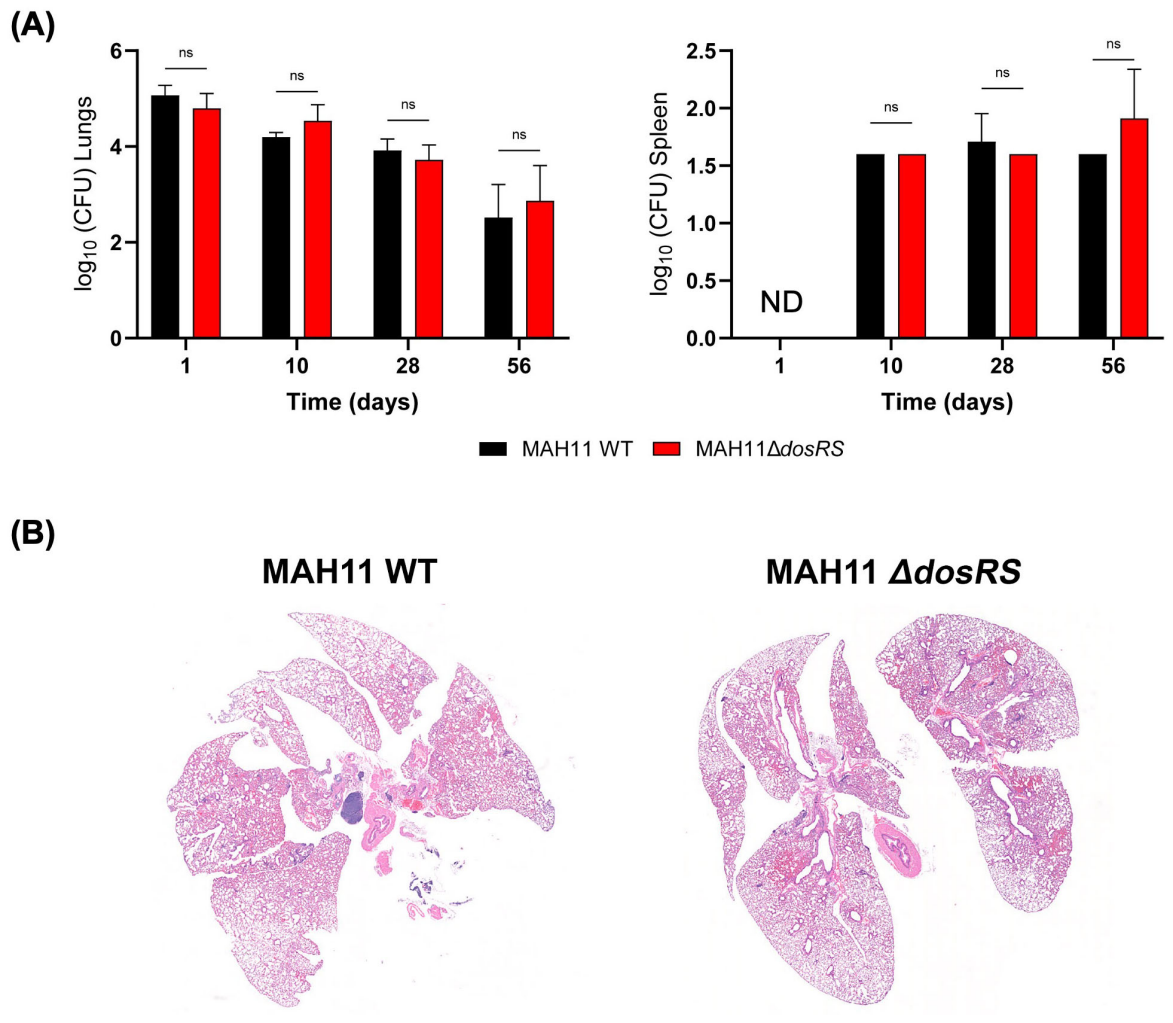
Infection of BALB/c mice with WT *M. avium* MAH11 and the *dosRS* knock-out mutant. **(A)** BALB/c mice were infected intratracheally with 1.0x10^6^ CFU of either MAH11 WT or MAH11Δ*dosRS*. Groups of mice were euthanized 1, 10, 28 and 56 days post-infection, and lungs and spleens were taken for bacterial enumeration (CFU). For each bacterial strain, the CFU represent the average of 5 mice per time point, and bacterial loads are expressed as log_10_ CFU ± SD. ND, not determined. “ns” indicates that the difference between the two strains is not significant pursuant to two-way ANOVA. **(B)** Representative histopathology (on day 56) of mice infected with MAH11 WT or MAH11Δ*dosRS* is shown. Subgross, low magnification images of total lung tissue are shown. Inflammatory pathology is rare and extent of disease is not different between WT- and Δ*dosRS*-infected mice.

The contribution of the DosRS two-component regulator to virulence and pathogenesis was next studied using a BALB/c mouse model of MAC infection. BALB/c mice were infected intratracheally with WT MAH11 and the *dosRS* knock-out mutant. Over the 56 days of infection, the bacterial burden in the lungs and spleen of WT- and Δ*dosRS*-infected animals remained comparable, slightly decreasing over time (**Figure 5A**). Limited disease progression was observed by histopathology regardless of infection with WT or Δ*dosRS*. Rare areas of inflammation contained a mixture of macrophages and lymphocytes with fewer neutrophils. Across all animals, inflammatory pathology affected less than 2% of the total lung tissue area, indicating a low burden of pulmonary pathology that was comparable between the two strains (**Figure 5B**).

## 4 Discussion

Homologs of DosRS regulon genes have been identified not just in the genomes of pathogenic mycobacteria but also in that of environmental mycobacteria as well as other environmental prokaryotes and archaebacteria (Bartek et al., 2009; Gerasimova et al., 2011; Selvaraj et al., 2012). This broad distribution has long suggested that DosR regulons did not primarily evolve for pathogenesis or drug tolerance but rather for adaptation to anaerobic conditions within the environment, and has been adapted by *Mtb* and other mycobacterial pathogens of the human lung for survival inside activated macrophages and in the avascular necrotic regions of granulomas. Accordingly, the opportunistic NTM, *M. avium*, responded to a shift from normoxic to microaerophilic conditions by a strong (> 4-fold) upregulation of 16 genes, 13 of which appeared to require DosRS for induction (**Figure 2B**). The existence of a DosR-binding motif in the promoter region of 10 of these 13 genes indicates that greater than 75% of them are likely under the direct transcriptional control of DosR, thereby implicating the two-component regulatory system DosRS as a major driver of the early response of *M. avium* to hypoxia. The fact that the upregulation of these 13 genes upon oxygen depletion required both *dosR* and *dosS* (complementation of the *dosRS* mutant with *dosR* alone failed) establishes DosS as the sole and essential sensor kinase involved in this response (**Table 1**).

A bioinformatic study predicted a 12-gene DosRS regulon in *M. avium* 104 and an 8-gene regulon in *M. abscessus*, the smallest regulons of all mycobacterial species analyzed to date (Gerasimova et al., 2011). Comparatively, the *Mtb* DosRS regulon comprises 48 genes (Rustad et al., 2008) and that of *M. marinum* is predicted to contain 66 (Gerasimova et al., 2011). Our RNAseq results confirm the relatively small size of the *M. avium* DosRS regulon and validate the DosRS-dependent expression of 10 of the 12 genes predicted bioinformatically by Gerasimova et al. Comparing the 13 *M. avium* DosRS-dependent genes induced under microaerophilic conditions identified in this study (**Figure 2B**; **Table 1**) to the DosRS regulons of *Mtb* and *M. abscessus*, 6 and 4 homologs were shared between species, respectively, with three genes (*MAV_2505* [*acg*], *MAV_2507* and *MAV_4010*) being found in all three genomes (**Table 1**). These genes encode an NAD(P)H nitroreductase (Acg) and two putative universal stress proteins, respectively, and are part of the minimal dormancy regulon defined by Gerasimova et al. (2011). The other 10 *M. avium* DosRS-dependent genes induced under microaerophilic conditions share similar functions with genes reported earlier to be induced under microaerophilic conditions and/or upon exposure to NO in *Mtb* and *M. abscessus* (Voskuil et al., 2003; Rustad et al., 2008; Simcox et al., 2023). They encode a putative fatty acid desaturase, a sensor kinase-like protein (MAV_2508), a truncated hemoglobin potentially involved in protection against NO, a pyridoxamine 5’-phosphate oxidase involved in the recycling of enzymatic cofactors, two additional nitroreductases potentially involved in protection against nitrogen stress, two additional universal stress proteins, and two hypothetical proteins of unknown function.

Like *Mtb*, *M. avium* has the ability to persist in a non-replicating state inside foamy macrophages that contain abundant lipid bodies (Caire-Brandli et al., 2014). *M. avium* residing within these cells forms intracytoplasmic triglyceride inclusions that provide a source of nutrients and energy facilitating persistence or bacterial regrowth. In *Mtb*, triglyceride build-up has been associated with non-replicating persistence and drug tolerance, and is largely controlled by *tgs1*, a triglyceride synthase gene under control of DosR (Baek et al., 2011). The comparable production of triglycerides by WT MAH11 and MAH11Δ*dosRS* grown under microaerophilic conditions (**Supplementary Figure S2**), and apparent lack of regulation of any of the 10 *M. avium* triglyceride synthase genes by DosR (**Figure 2A**) point to an alternative mechanism of control of triglyceride synthesis in this species.

In line with the nature of the *M. avium* DosRS regulon, the ability of MAH11Δ*dosRS* to survive in the Wayne hypoxic model was significantly reduced (**Figure 3A**). Lowering the pH from 7.3 to 5.7 further decreased survival, presumably due to the compromised ability of the mutant to maintain cytosolic pH homeostasis (Reichlen et al., 2017). Despite the fitness advantage conferred by DosRS in hypoxia, loss of DosRS had no significant impact on the ability of *M. avium* to form biofilms in host-relevant SCFM medium. This result is in contrast to observations made in *M. abscessus* (Belardinelli et al., 2022) and suggests that *M. avium* has evolved alternative mechanisms to support the growth and survival of bacilli within the hypoxic environment of biofilms. Loss of DosRS also did not significantly impact the tolerance of *M. avium* MAH11 to clinically used antibiotics in the Wayne model, similar to the situation in *Mtb* (another slow-growing *Mycobacterium*) (Bartek et al., 2009) but opposite to that in the fast-growing species, *M. abscessus* (Belardinelli et al., 2022). A noticeable exception was that of nitrofurans to which the *dosRS* mutant became significantly more susceptible (**Figure 3E**). Nitrofurans, like nitroimidazoles such as metronidazole, are prodrugs that require NAD(P)H-dependent nitroreductase activity under anaerobic conditions to reduce their nitro or nitroso groups and release bactericidal intermediates. Some microbial NAD(P)H-dependent nitroreductases, however, inactivate nitro(so) drugs by fully reducing them to non-toxic forms of the antibiotic (Muller et al., 2013; Saghaug et al., 2023). The hypersusceptibility to nitrofurans of the MAH11Δ*dosRS* mutant (which has lost the ability to upregulate three putative nitroreductases under hypoxia) suggests that one or more of these enzymes have the ability to inactivate nitrofurans. *M. avium* DosRS-dependent nitroreductases, however, do not appear to confer any protective effect against metronidazole (**Figure 3E**).

Despite being induced upon macrophage infection, the DosRS regulon is not critical to the survival of *M. avium* MAH11 inside macrophages under the conditions of this study (**Figure 4**). It further did not confer any survival or pathogenic advantage in infected BALB/c mice over two months of infection (**Figure 5**). The fact that only one clinical isolate of *M. avium* was tested therein combined with the low pathogenicity of MAH11 in murine models of infection and the fact that BALB/c mice do not form necrotic hypoxic granulomas are limitations of this study. It is possible that the MAH11Δ*dosRS* mutant might only display an attenuation phenotype in animal models of infection that develop hypoxic caseous necrotic granulomas. This situation indeed has precedence with *Mtb* where *dosR* mutants only presented attenuation phenotypes in rabbit, guinea pig and nonhuman primate models of infection (Zheng et al., 2020). Whether the same conclusion applies to *M. avium* will have to await the development of models of infection mimicking the human pathology for this NTM pathogen.

## Data Availability

The data presented in the study are deposited in the NCBI Sequence Read Archive (SRA) under accession number PRJNA1191895. https://www.ncbi.nlm.nih.gov/bioproject/PRJNA1191895.
