# Supplementary material for: The DosR regulon of Mycobacterium avium and adaptation to hypoxia
Source: Front Cell Infect Microbiol. 2025 Feb 18;15:1545856. doi: 10.3389/fcimb.2025.1545856 (PMC11876180; doi:10.3389/fcimb.2025.1545856)

SUPPLEMENTARY MATERIALS

The DosR regulon of *Mycobacterium avium* and adaptation to hypoxia

Juan M. Belardinelli[1], Charlotte Avanzi[1], Kelsey E. Martin[1,2], Ha Lam[1], Marte Dragset[3,4], William H. Wheat[1], Brendan K. Podell[1], Mercedes Gonzalez-Juarrero[1,2], Mary Jackson[1,2*]

[1]Mycobacteria Research Laboratories, Department of Microbiology, Immunology and Pathology, Colorado State University, Fort Collins, CO, USA; [2]Cell and Molecular Biology, Colorado State University, Fort Collins, CO, USA; [3]Centre of Molecular Inflammation Research, Norwegian University of Science and Technology, Trondheim, Norway; [4]Department of Clinical and Molecular Medicine, Norwegian University of Science and Technology, Trondheim, Norway

Supplementary Figures

Figure S1: Generation of a *dosRS* knock-out mutants of *M. avium* MAH11.

Figure S2: Effect of knocking-out *dosRS* on the biosynthesis of triglycerides.

Figure S3: *MAV_2508* is not required for the persistence of *M. avium* MAH11 to hypoxia.

Figure S4: Susceptibility of *M. abscessus* ATCC 19977 to NO.

<u>Figure S1</u>: Generation of a *dosRS* knock-out mutants of *M. avium* MAH11.
Gene replacement at the *dosRS* loci of *M. avium* MAH11 was achieved using the Ts/sacB method and confirmed by PCR using sets of primers located outside the allelic exchange substrate. The expected sizes of the PCR products for the WT and mutant strains are 3,623 bp and ~ 2,500 bp, respectively. MWM, molecular weight marker.

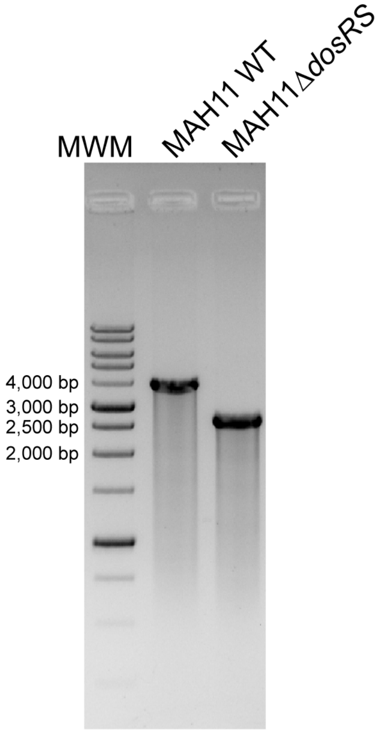

<u>Figure S2</u>: Effect of knocking-out *dosRS* on the biosynthesis of triglycerides.

Cultures of *M. avium* MAH11 WT, MAH11Δ*dosRS* and MAH11Δ*dosRS* complemented with *dosR* or *dosRS* grown in Dubos-Tween albumin broth under well-aerated (oxygenated) or in standing T25 vented flasks (microaerophilic) were labeled with [1,2-^{14}C]acetic acid for 16 h. Extracted lipids from each strain (10,000 cpm per lane) were analyzed by TLC using hexane:diethyl ether:acetic acid (70:30:1 by vol.) as the eluent and revealed by PhosphorImaging. TAG, triglycerides; DAG, diglycerides.

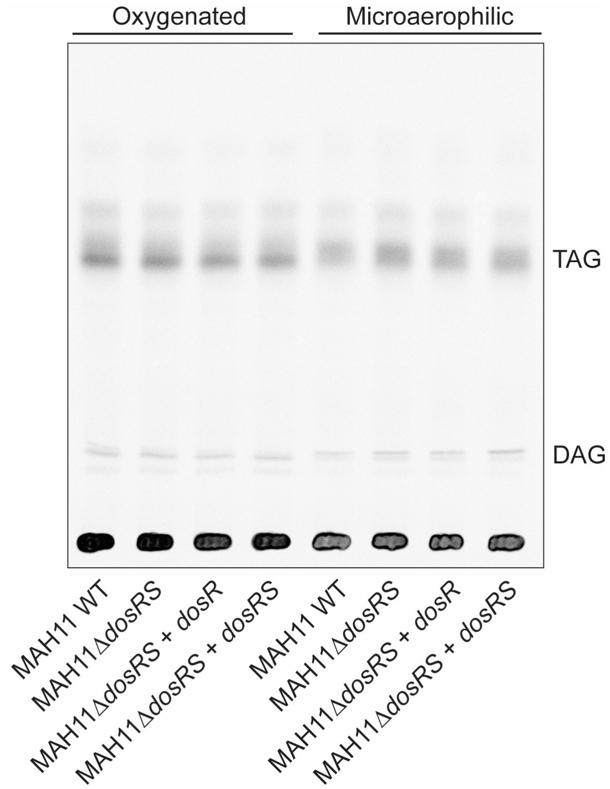

<u>Figure S3</u>: *MAV_2508* is not required for the persistence of *M. avium* MAH11 to hypoxia. Comparative survival curves of MAH11 WT and mutants carrying transposon insertions at bp 220 (of 1413 bp) of the *MAV_2508* gene or at position 549 (of 669 bp) of *dosR* (*MAV_4109*) in the Wayne model at pH 7.3. At the indicated time points, tubes were opened and serial dilutions of the cultures plated for CFU enumeration. The results presented are the means (± SD) of technical triplicates. Asterisks denote statistically significant differences relative to WT MAH11 pursuant to one-way ANOVA, with *p<0.005; ***p<0.0005; and ****p<0.0001; ns, not significant. The results are representative of two independent experiments.

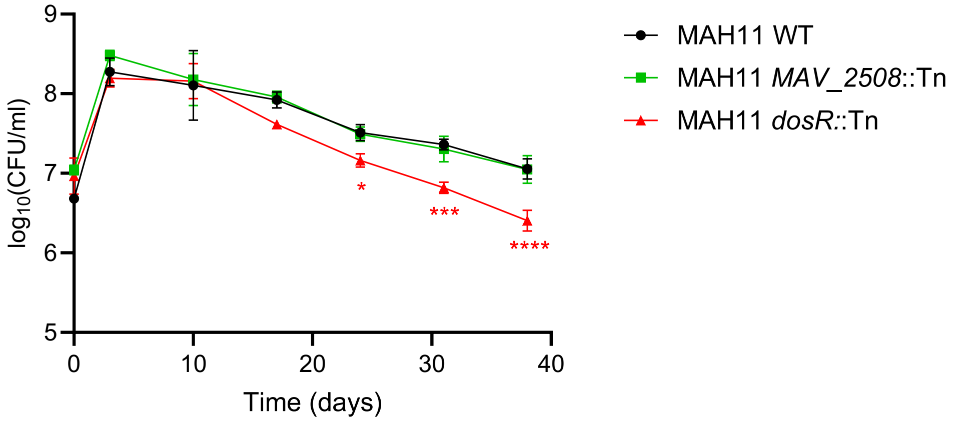

<u>Figure S4</u>: Susceptibility of *M. abscessus* ATCC 19977 to NO.
Triplicate cultures of *M. abscessus* ATCC 19977 (smooth morphotype) grown in 7H9-ADS-Tween 80 were treated with 50 or 500 μM DETA/NO or Spermine/NO for 24 h and subsequently plated for CFU enumeration.

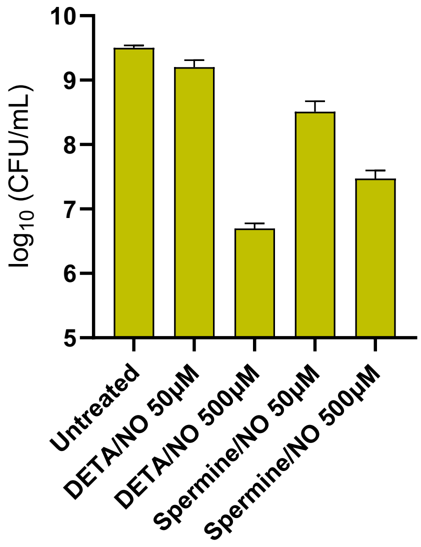

Supplement: Supplementary file 1 [file DataSheet1.pdf]
